# Supplementary material for: The Role of Bifidobacterium in Liver Diseases: A Systematic Review of Next-Generation Sequencing Studies
Source: Microorganisms. 2023 Dec 17;11(12):2999. doi: 10.3390/microorganisms11122999 (PMC10745637; doi:10.3390/microorganisms11122999)
Supplement: Supplementary file 1 [file microorganisms-11-02999-s001.zip › Supplementary File 1.pdf]

Search strategy

PubMed - 183 (English, 2013-2023)

(Non-alcoholic Fatty Liver Disease[Mesh] OR NAFLD[tiab] OR NASH[tiab] MAFLD[tiab] OR Nonalcoholic Steatohepat*[tiab] OR Non-alcoholic Steatohepat*[tiab] OR Fatty Liver*[tiab] OR Hepatic Steatos*[tiab] OR Liver Diseases, Alcoholic[Mesh] OR Alcoholic Liver Disease*[tiab] OR Alcoholic Steatohepat*[tiab] OR Alcoholic Hepat*[tiab] OR Alcoholic Cirrhos*[tiab] OR Liver Cirrhosis[Mesh] OR Liver Cirrhos*[tiab] OR Hepatic Cirrhos*[tiab] OR Metabolic dysfunction-associated fatty liver disease*[tiab] OR Metabolic associated fatty liver disease*[tiab] OR Metabolic-associated fatty liver disease*[tiab] OR Carcinoma, Hepatocellular[Mesh] OR Hepatocellular Carcinoma*[tiab] OR Hepatocellular Cancer*[tiab] OR HCC[tiab] OR Liver Cancer*[tiab] OR Liver Cell Carcinoma*[tiab])
AND
(Bifidobacterium*[tiab])

Embase – 192 (English, 2013-2023)

('nonalcoholic fatty liver'/exp OR 'alcohol liver disease'/exp OR 'liver cirrhosis'/de OR 'liver cell carcinoma'/exp OR (NAFLD OR NASH OR MAFLD OR 'Nonalcoholic Steatohepat*' OR 'Non-alcoholic Steatohepat*' OR 'Fatty Liver*' OR 'Hepatic Steatos*' OR 'Alcoholic Liver Disease*' OR 'Alcoholic Steatohepat*' OR 'Alcoholic Hepat*' OR 'Alcoholic Cirrhos*' OR 'Liver Cirrhos*' OR 'Hepatic Cirrhos*' OR ‘Metabolic dysfunction-associated fatty liver disease*’ OR ‘Metabolic associated fatty liver disease*’ OR ‘Metabolic-associated fatty liver disease*’ OR 'Hepatocellular Carcinoma*' OR 'Hepatocellular Cancer*' OR HCC OR 'Liver Cancer*' OR 'Liver Cell Carcinoma*'):ti,ab,kw)
AND
(Bifidobacterium*:ti,ab,kw)
AND
[embase]/lim NOT ([embase]/lim AND [medline]/lim)

Web of Science – 273 (English, 2013-2023)

TS=(NAFLD OR NASH OR MAFLD OR "Nonalcoholic Steatohepat*" OR "Non-alcoholic Steatohepat*" OR "Fatty Liver*" OR "Hepatic Steatos*" OR "Alcoholic Liver Disease*" OR "Alcoholic Steatohepat*" OR "Alcoholic Hepat*" OR "Alcoholic Cirrhos*" OR "Liver Cirrhos*" OR "Hepatic Cirrhos*" OR “Metabolic dysfunction-associated fatty liver disease*” OR “Metabolic associated fatty liver disease*” OR “Metabolic-associated fatty liver disease*” OR "Hepatocellular Carcinoma*" OR "Hepatocellular Cancer*" OR HCC OR "Liver Cancer*" OR "Liver Cell Carcinoma*")
AND
TS=(Bifidobacterium*)
